# P-1234. Evaluation of Empirically Dosed Parenteral Vancomycin Using Weight-Based Versus Bayesian-Based Modeling

**DOI:** 10.1093/ofid/ofae631.1416

**Published:** 2025-01-29

**Authors:** Julie Mei, Sheren Deyab, Luis Pereira, Rita Faour, Aliaksandr Budovich

**Affiliations:** One Brooklyn Health - Brookdale, Brooklyn, New York; One Brooklyn Health - Brookdale, Brooklyn, New York; One Brooklyn Health - Brookdale, Brooklyn, New York; One Brooklyn Health - Brookdale, Brooklyn, New York; One Brooklyn Health - Brookdale, Brooklyn, New York

## Abstract

**Background:**

Vancomycin is an efficacious antibiotic in treating infections caused by *Staphylococcus Aureus (including MRSA), streptococci, and enterococci*. Intravenous administration of vancomycin requires close monitoring to optimize safety and efficacy. As per ASHP/IDSA/SIDP guidelines, an empiric maintenance dose of 15-20 mg/kg (based on actual body weight) administered every 8-12 hours (based on normal renal function) with a target AUC of 400-600 is recommended for most adults. Given the lack of data comparing empiric Bayesian modeling to weight-based dosing of vancomycin, our study aims to evaluate the relationship between these two regimens.

**Methods:**

This is an IRB approved retrospective cohort study conducted at One Brooklyn Health Brookdale. A random sample of non-critically ill hospitalized patients aged 18 or older who have been treated with empirically dosed vancomycin (15-20 mg/kg) administered at the recommended frequency for suspected or documented infections between January 1, 2023 and December 31, 2023 will be included. Patients will be excluded if they have acute kidney injury (AKI), are on hemodialysis, or have a BMI >30 kg/m^2^. Pertinent patient data will be collected such as patient demographics, laboratory values and etc. Statistical analysis will be conducted using SAS statistical software.

**Results:**

A total of 97 patients met inclusion criteria. Primary outcome was defined as the difference in numbers of patients achieving AUC 400-600 using empiric weight-based versus Bayesian modeling dosing. Patients were about 26% more likely to have a therapeutic AUC when being dosed on a weight-based regimen. Secondary outcomes looked at the correlation between weight and weight-based dosing and Bayesian modeling dosing in terms of subtherapeutic or supratherapeutic AUC and found that there was no association. Safety outcome was defined as the difference in number of patients at an increased risk of AKI (AUC > 650) using empiric weight-based versus Bayesian modeling dosing. Patients were 50% less likely to be at an increased risk of AKI when being dosed on the weight-based regimen.

**Conclusion:**

Bayesian-based modeling for empiric dosing was associated with higher rates of supra-therapeutic AUC and individuals were at a higher risk of getting AKI.

**Disclosures:**

**All Authors**: No reported disclosures

